# Detection of mild cognitive impairment in people older than 65 years of age and its relationship to cardiovascular risk factors (DECRIVAM)

**DOI:** 10.1186/1471-2458-11-504

**Published:** 2011-06-27

**Authors:** M Victoria Perea-Bartolome, Ricardo García-García, Valentina Ladera-Fernández, Sara Mora-Simón, María C Patino-Alonso, Tita J Almanza-Guerra, Cristina Agudo-Conde, M Paz Muriel-Diez, Emiliano Rodríguez-Sánchez

**Affiliations:** 1 Departamento de Psicología Básica, Psicobiología y Metodología de las Ciencias del Comportamiento. Facultad de Psicología. Universidad de Salamanca. Salamanca, Spain; 2 Primary care research unit of La Alamedilla Health Center, Castilla y León Health Service - SACYL. Salamanca, Spain; 3 Departamento de Estadística. Universidad de Salamanca. Salamanca, Spain

## Abstract

**Background:**

Studies centered on the detection of cognitive impairment and its relationship to cardiovascular risk factors in elderly people have gained special relevance in recent years. Knowledge of the cardiovascular risk factors that may be associated to cognitive impairment could be very useful for introducing treatments in early stages - thereby possibly contributing to improve patient quality of life.

The present study explores cognitive performance in people over 65 years of age in Salamanca (Spain), with special emphasis on the identification of early symptoms of cognitive impairment, with the purpose of detecting mild cognitive impairment and of studying the relationships between this clinical situation and cardiovascular risk factors.

**Methods/Design:**

A longitudinal study is contemplated. The reference population will consist of 420 people over 65 years of age enrolled through randomized sampling stratified by healthcare area, and who previously participated in another study. Measurement: a) Sociodemographic variables; b) Cardiovascular risk factors; c) Comorbidity; d) Functional level for daily life activities; and e) Study of higher cognitive functions based on a neuropsychological battery especially adapted to the evaluation of elderly people.

**Discussion:**

We hope that this study will afford objective information on the representative prevalence of cognitive impairment in the population over 65 years of age in Salamanca. We also hope to obtain data on the relationship between cognitive impairment and cardiovascular risk factors in this specific population group. Based on the results obtained, we also will be able to establish the usefulness of some of the screening tests applied during the study, such as the Mini-Mental State Examination and the 7 Minute Screen test.

**Trial registration:**

ClinicalTrials.gov: NCT01327196

## Background

The study of cognitive impairment in elderly people presently constitutes an important challenge, considering that the population life expectancy has increased significantly in the last decades. With advancing age, and particularly after 60 years of age, the prevalence of cognitive impairment increases almost exponentially, and represents one of the main causes of disability among the elderly[1-5]. In the European Union it has been estimated that over 5 million inhabitants suffered dementia in the year 2005 (1.14-1.27% of the population), with a total of 583,208 registered cases in Spain that year [6]. In the region of Castilla y León, the estimated prevalence of dementia is 6.4% in people aged 65 years and older - representing about 43,500 individuals. This situation now constitutes an important clinical, healthcare and socioeconomic problem, in view of the sanitary and care requirements involved, the important family impact, and the scarcity of effective therapeutic resources. Few studies on the economical cost of dementia have been conducted in our setting, though the estimated cost exceeds 9000 euros per patient and year, considering only the direct healthcare costs: drugs, diagnostic studies, medical visits, etc. If the indirect costs associated with the disease are also taken into account, then the figure per patient reaches 19,000 euros per year. During the year 2004, the cost totaled over 8200 million euros in Spain alone [7].

Although cognitive impairment is the most relevant symptom, dementia is usually also associated to different behavioral, psychological and functional manifestations that affect the functional and executive capacities of the individual - interfering with normal family, social and occupational activities, and lessening the quality of life of both the patients and their caregivers. Dementia is one of the main causes of early patient institutionalization. It has been estimated that behavioral or neuropsychiatric symptoms can manifest in 50% of all elderly patients with dementia, and the frequency is moreover higher among institutionalized individuals [4,8]. These symptoms can show diverse presentations, in any evolutive stage of the disease, and typically manifest in multiple and oscillating forms [4].

At present, the most common cause of dementia is Alzheimer's disease (AD), of probable multifactorial origin (genetic, environmental, toxic, etc.), followed by vascular dementia (VD). The role of cerebrovascular disease as a cause of dementia has been recognized for over a century. We are now witnessing a redefinition of the different forms of VD, with introduction of the concept of vascular cognitive impairment, designed to identify the early stages of a process hypothetically amenable to preventive intervention and treatment. Together with the classical vascular lesions (arterial and lacunar infarctions), other types of cerebrovascular lesions are becoming known such as leukoaraiosis or incomplete infarctions, which contribute to the development of intellectual impairment, and increased insight is being gained to the clinical, neuropsychological and radiological correlations involved. Behavioral and psychological manifestations are increasingly investigated and recognized as symptoms inherent to VD [9,10]. Descriptive, analytical and interventional epidemiological studies have underscored the relevance of vascular factors as causal agents in dementia [11,12]. Another important epidemiological aspect in the field of vascular cognitive impairment refers to cognitive impairment presenting in stroke patients. Up to 30% of all patients with cerebral infarction develop dementia in the three months following stroke - this representing a 9-fold greater risk versus the controls without stroke [12]. The repetition of stroke may trigger dementia in some but not in all cases, and many patients develop dementia without any apparent underlying clinical event. Furthermore, in many cases the observed dementia corresponds to Alzheimer's disease instead of vascular dementia. This point is important if the vascular component of mixed dementia is amenable to preventive treatment.

Different strategies have been proposed for the prevention of dementia, though none have yielded positive results. Given the absence of quality clinical evidence supporting their use, drug treatments (statins, hormone replacement therapy or nonsteroidal antiinflammatory drugs (NSAIDs)) are not advised for the primary prevention of dementia [11]. This discouraging reality is applicable to most dementias, since these are due to Alzheimer's disease, but it is possible to prevent the second most common cause of dementia, i.e., vascular dementia [1,3,4], which manifests as a consequence of cerebrovascular disease. Regarding the primary prevention of dementia, the PAPPS study [13] recommends remaining socially and mentally active, physical activity, and the observation of healthy living habits, with adequate control of cardiovascular risk factors - particularly correct blood pressure control.

Cardiovascular diseases are the main cause of death in Spain, and are attributable particularly to cerebrovascular disorders and ischemic heart disease.

Arterial hypertension is a systemic condition that preferentially affects three body organs: the heart, kidneys and brain. In contrast to the heart and kidneys, which are systematically evaluated in clinical practice, brain function is often neglected. Hypertension and dementia are common disorders in the population over 65 years of age. It has been estimated that 8% and 65% of these individuals suffer dementia and hypertension, respectively [14]. From this age onwards, the prevalence of dementia increases exponentially, doubling every 5 years and reaching 28.5% in people aged 90 years or older (EURODEM study)[15,16].

The results of epidemiological studies in reference to the association between these two disorders vary depending on the methodology employed [17]. Thus, while cross-sectional studies have reported contradictory results [18-21], longitudinal surveys point to a positive relationship between the presence of arterial hypertension in middle age and mild cognitive impairment 15-20 years later [22-24]. No such association has been observed when arterial hypertension develops at an older age [25-27]. Skoog et al. [28] found arterial hypertension at 70 years of age to be greater in subjects who developed dementia between 79 and 85 years of age. However, during the years preceding the onset of dementia, blood pressure decreases in comparison with those subjects who do not develop dementia. Similar findings have been published by Hanon [29] and Verghese [30]. The correlation between blood pressure and mild cognitive impairment is more complex than a simple lineal relationship. Chronic hypertension predisposes to mild cognitive impairment and the development of dementia, but a drop in blood pressure can be observed in the phase prior to the development of diffuse cognitive impairment [28].

Likewise, no clear effect of antihypertensive treatment has been observed in terms of the prevention of mild cognitive impairment in hypertensive patients. A Cochrane review [31] concludes that "*There was no convincing evidence in the reviewed trials to suggest that lowered blood pressure prevents the development of dementia or cognitive impairment in hypertensive patients without manifest prior cerebrovascular disease". *However, on considering the evidence of the cross-sectional, longitudinal and observational studies together with the evidence generated by the randomized clinical trials (RCTs), moderately solid support is found of the notion that hypertension in adult life - particularly when not treated effectively - exerts a negative effect upon cognition, and contributes to the development of dementia and Alzheimer's disease in old age [17,19,32]. There is less evidence from these studies that the same negative effect upon cognition is present with hypertension at an older stage in life [19].

The relationship between cholesterol and mild cognitive impairment is contradictory. There are several cross-sectional PAPPS studies with differing results [33,34]. There are also several longitudinal studies reporting a positive association between total cholesterol and mild cognitive impairment, while three observe no association, and one reports a negative association [35].

In reference to diabetes mellitus, a number of studies have reported an association with the prevalence of dementia, though others have found no such relationship. Biessesls et al. [36], following an analysis of several longitudinal studies, found the incidence of dementia to increase between 50-100% in subjects with diabetes versus individuals without diabetes. Associations have also been described with blood glucose [37] and glycosylated hemoglobin. In this sense, for every 1% rise in HbA1c, an increased risk of cognitive impairment or dementia has been reported (OR 1.40: CI 1.08-1.83)[38].

Metabolic syndrome groups a series of cardiovascular risk factors; as a result, an increased risk of target organ damage could be expected in patients with this syndrome. However, few studies have evaluated the association between metabolic syndrome and the development of dementia or mild cognitive impairment. Some cross-sectional [39] and longitudinal studies [40] have described an association between metabolic syndrome and cognitive impairment. Thus, while a certain relationship appears to exist, the risk of dementia in individuals with metabolic syndrome has not been firmly established, and it is not clear whether such risk is greater than the sum of its components [38].

Comorbidity among people over age 65 is common and conditions the prognosis of other concomitant disease processes, with an acceleration of the possible complications. Different methods are available for assessing comorbidity. Charlson proposed a numerical scale using a list of pathological conditions: the Charlson comorbidity index [41,42]. This index was designed to classify comorbidities for prognostic purposes, and posteriorly has been used as a control of the influence which comorbidity may exert in different situations. The Charlson index has not been used in the analysis of cardiovascular risk factors and cognitive impairment. Likewise, no study has been made of the association of cardiovascular risk as estimated from the risk scales and cognitive impairment in individuals over 65 years of age.

Less than one-half of all patients with Alzheimer's disease have been diagnosed, and of these subjects, approximately 25% are receiving some kind of treatment [43]. The number of individuals with undiagnosed cognitive impairment is therefore expected to be high, particularly in groups with certain risk factors. One of the factors explaining why many people with cognitive impairment have not been diagnosed is related to the fact that we have no cognitive impairment screening tool in the elderly population sufficiently warranted by the scientific community and the professionals who care for such patients.

Based on a neuropsychological battery especially adapted for the study of elderly people, we will carry out a study of cognitive performance in people over 65 years of age in Salamanca (Spain), with special emphasis on the identification of early symptoms of cognitive impairment, with the purpose of detecting mild cognitive impairment and of studying the relationships between this clinical situation and cardiovascular risk factors.

The following objectives have been established:

1. To study the cognitive performance of people over 65 years of age in Salamanca, with a view to estimating the prevalence of the different manifestations of cognitive impairment and its severity.

2. To analyze the association of cardiovascular risk factors (diabetes, arterial hypertension, dyslipidemia and metabolic syndrome) in patients over age 65 with mild cognitive impairment.

3. To use the Mini - Mental State Examination (MMSE) and the 7 Minute Screen test as screening tools for cognitive impairment in the elderly population under study.

## Methods/Design

### Design and study population

A longitudinal study will be carried out, since we will be starting from a reference population of 327 people over 65 years of age living in the city of Salamanca (Spain) and who participated in the cross-sectional study entitled "Sociosanitary needs of people over 65 years of age in the city of Salamanca", financed by the regional health authorities (SOCIO673/01/08). Randomized sampling is contemplated, stratifying for healthcare area.

#### Power analysis

The population universe was the city of Salamanca, with a total of 172,375 people. A 19.74% out of them (34,020) was 65 years old or more, while in rural emplacement of the region the population older than 65 years old was a 30.25%. It was estimated that it would be a 16% at least.

Taking an alpha risk of 0.05, a power of 20% and estimating the sum of prevalence of mild cognitive impairment plus dementia around a 16%, with a mistake of 4%, and taking into consideration the current population of 65 years old or older, it would be needed 320 participants. Considering up to a 50% of loss for no response and according to similar studies, sample size was 480 participants.

In this first study the population structure was taken into account as established from the individual healthcare cards database of the Primary Care management authorities of Salamanca, with sampling stratified according to healthcare centers. The mentioned study was carried out through home interviews.

### Measurements

The principal study measurements are the following:

a) Sociodemographic variables: age, sex, educational level, marital status, family unit.

b) Anthropometric variables and physical evaluation: body weight, height, waist circumference, blood pressure, heart rate, physical activity, lipid profile, blood glucose.

c) Vascular risk factors: smoking, hypertension, diabetes mellitus, dyslipidemia, obesity, alcohol intake.

d) Cardiovascular disease: stroke, heart disease, neuropathy, retinopathy.

e) Drug use (antihypertensive, antidiabetic, lipid-lowering and antiplatelet drugs, psychoactive medication).

f) Comorbidity.

g) Functional grade will be evaluated in reference to daily life activities (both basic and instrumental), and data from an informant will be obtained.

h) Neuropsychological assessment: assessment of the premorbid situation, attention-concentration, speech, logical memory, auditory-verbal learning, visioconstruction memory, visual memory, executive function. Following evaluation, the impairment phases will be classified, ranging from normality to the most severe phases of dementia.

### Measurement instruments

#### 1. Anthropometric measurements

a) Height: the mean of two measurements using a wall meter (Seca 222), with the patient barefoot and in the erect standing position, and measuring to the nearest millimeter. Body mass index: weight (kg)/height (m)^2^.

b) Waist circumference: the mean of three measurements, using a flexible measuring tape at waist level (at the midpoint between the last rib and the iliac crest), parallel to the floor, and measuring after inspiration.

c) Weight: the mean of two measurements using a homologated and easily calibrated scale (Seca 770) with the patient barefoot and lightly clothed. The readings will be rounded to 100 grams.

d) Clinical blood pressure (OMROM M-7): three measurements of systolic blood pressure (SBP) and diastolic blood pressure (DBP) will be made, calculating the mean of the last two readings, and using a validated sphygmomanometer (OMRON M7), following the recommendations of the European Society of Hypertension. Pulse pressure (PP) will be estimated with the mean values of the second and third measurements.

e) Physical activity: Physical activity will be estimated by the 7-day Physical Activity Recall (PAR)[44]. The PAR is a general measure of physical activity, which has been recognized as a valid and reliable tool in recent years, and is widely used in epidemiological, clinical and behavioral change studies. It consists of a semi-structured interview (10-15 minutes) in which the participants provide an estimate of the number of hours dedicated to physical or occupational activities requiring at least a moderate effort in the past 7 days. The categories collected are: moderate, vigorous, and very vigorous physical activity. The dose of physical exercise will be estimated in METs/hour/week, and active persons will be taken to be those doing at least 30 minutes of moderate activity, 5 days a week, or at least 20 minutes of vigorous activity, 3 days a week. Persons not reaching this level of physical activity are regarded as sedentary.

#### 2. Cardiovascular risk and stroke risk scales

Estimated risk at 10 years will be calculated according to the Framingham risk scales for stroke and global cardiovascular morbidity-mortality [22].

#### 3. Measurement of comorbidity

The disorders established by Charlson in the original article published in 1987 [41] are: myocardial infarction; congestive heart failure; arterial hypertension; peripheral vascular disease; cerebrovascular disease; dementia; chronic pulmonary disease; connective tissue disease; peptic ulcer; mild, moderate or severe liver disease; diabetes with or without organ damage; hemiplegia; leukemia; lymphoma; solid tumor with metastasis; AIDS; and moderate or severe kidney disease. After establishing patient comorbidity, each entity will be scored in a table - the sum of the scores representing the Charlson comorbidity index (CCI). In order to calculate the age-adjusted Charlson comorbidity index (ACCI), we add one point to the CCI for each decade above 50 years of age. The values obtained will be grouped into four categories: 1-2, 3-4, 5-7 or ≥ 8.

#### 4. Need for help in daily life activities

Use will be made of the Barthel index [45] for the evaluation of basic daily life activities and of the Lawton and Brody index [46] for the evaluation of instrumental daily life activities. The short version informant test (IQCODE) will be applied [47,48]. This is a questionnaire to be completed by a relative or person close to the patient. Following a series of short instructions, and in very little time, the informant is asked to recall how the patient was 10 years ago, and how he or she has changed since then, based on a 5-point Likert scale. The short version comprises 17 questions.

#### 5. Neuropsychological assessment protocol

a) Cognitive screening: The Folstein Mini-Mental Test, MMSE [49], and the 7 Minute Screen [50,51], are cognitive screening tests that explore a number of cognitive areas which are particularly affected in an early stage in Alzheimer-type dementia and in other dementias.

b) Evaluation of the premorbid situation: The word accentuation test [52] allows us to quickly estimate the premorbid intellectual level of the subject. It consists of 30 infrequent words that are presented to the subject (without accentuations), and which he or she must read correctly (phonetic accent).

c) Attention-concentration: This is a digit subtest to the right and reverse [53]. It assesses attention, as well as storage and immediate recall of a series of numbers.

d) Speech: The Boston vocabulary test [54] explores denomination capacity based on the visual confrontation of 60 images.

Semantic - phonetic generation [55], comprising two subtests. The first, *names of animals*, evaluates semantic verbal fluency. The subject is asked to name animals as quickly as possible and in one minute. The score is the number of words evoked by the patient. The second subtest is *words that begin with the letter "p"*, and evaluates phonologic verbal fluency. The subject is asked to name words that begin with the letter "p". The score is the number of words evoked by the patient in a period of one minute.

e) Logical memory: Babcock story [56], immediate and delayed. The explorer reads a story to the subject, who in turn is required to recall it immediately, thereby assessing retention memory and immediate recall corresponding to logical structured verbal material. After non-mnesic interference (approximately 15 minutes), the subject is instructed to repeat what he or she remembers about the story (delayed logical verbal memory). The story consists of 21 information units.

f) Visuoconstruction memory: Rey complex figure test [57]. This test involves two phases: copy and memory. Memory reproduction informs us of the visual memory of the subject, as well as of his or her visuoconstruction capacity. Subject performance is evaluated according to the number of correct units conforming the complete figure (the maximum score being 36), the type of execution of the subject, and the time (in seconds) used to construct the figure.

h) Visual memory: The Benton visual retention test will be used [58]. We will apply form C, Administration A. This involves presentation of each of the sheets, during 10 seconds, followed by immediate memory reproduction or recall by the subject. This test evaluates the visuospatial perception of the subject, as well as visual memory, visuoconstruction skills and visual conceptualization. Subject performance is evaluated according to the number of correct reproductions (the maximum score being 10) and error assessment (taking into account the specific type of error made by the subject: omission, distortion, displacement, perseverance, rotation, size and whether located to left or right).

i) Executive function: Abbreviated Frontal Assessment Battery (FAB)[59]. This test evaluates frontal lobe functions based on 6 tasks: similarities (formation of concepts), verbal fluency (mental flexibility), motor series (programming), interference (execution of conflictive instructions), control (response inhibition) and autonomy (environmental independence). The action-verb fluency test [60] in turn requires the subject to evoke the largest possible number of words designating an action (verbs), in 60 seconds. The score in this task is the total number of words correctly evoked within the limited time interval.

g) Visuoconstruction skills: the Rey complex figure test [57]. Copying of the figure evaluates subject visual perception, practical-constructive skills, visuospatial analytical capacity, and right-left orientation. The assessment is the same as commented above in the memory figure reproduction phase.

h) Cognitive impairment: classification of the severity of cognitive impairment in 7 global stages ranging from normal to severe impairment, based on the Global Deterioration Scale (GDS) [61].

### Procedure

All participants will be informed of the purpose of the study by telephone and conventional mail, and will be asked to give written informed consent to participation. Once consent has been obtained, all subjects will receive a card stating the place, date and exact time of the evaluation. One day before the study the subjects will be telephoned to remind them of the appointment. Evaluation will be carried out in the Faculty of Psychology of the University of Salamanca, in Room 19 (specifically used for neuropsychological tests).

The sessions will be individualized, and the procedure will be the same for all participants, using the aforementioned measurement instruments.

The estimated duration of the evaluation is 120 minutes, with two resting intervals of about 15 minutes each.

The clinical and laboratory test data can be collected on occasion of a second interview to be arranged with the participants and/or relatives.

### Statistical analysis

A descriptive analysis of the data will be made, establishing comparisons with the baseline evaluation of the previous study. Posteriorly, an analysis will be made of the qualitative variables using the chi-squared test, and the qualitative and quantitative variables will be analyzed using the Student t-test and analysis of variance (ANOVA), depending on whether two or more categories are involved. An analysis will be made of the correlations of the quantitative variables, together with multiple regression analysis to identify the variables associated with cognitive impairment diagnosed by the neuropsychological tests.

Likewise, an evaluation will be made of the sensitivity, specificity and predictive values of the tests employed in screening for cognitive impairment, using the neuropsychological evaluation made as gold standard.

Lastly, we will analyze the evolution of the different parameters between the two evaluations made, attempting to identify those variables with greater predictive capacity in reference to the evolution of cognitive impairment.

The information will be analyzed according to the place of residency, age and sex of the patient, the family situation and patient cognitive impairment, risk factors and comorbidity index.

Data input will be made using the Teleform system, with a questionnaire previously designed for the project, and exporting the data to the SPSS statistical package for posterior analysis.

### Ethical and legal issues

In order to guarantee data confidentiality, all the electronic and paper copies of the protocol, signed informed consent documents and results of the tests made in each of the patients will be kept locked in a safe place, and only the study investigators will have access to the data on the subjects who agree to participate in the study.

The protocol was approved (August 10, 2010) by the Clinical Research Ethics Committee of Salamanca University Hospital (Spain), and complies with Spanish data protection law 15/1999 and its recently developed specifications (Royal Decree (RD) 1720/2007). Knowledge and agreement to cooperate has been established with the implicated services, signed by the legal representative of the center.

### Methodological limitations

The main limitation is sample loss inherent to studies of this kind, since the voluntary nature of participation and the population characteristics referred to age, etc., implies a certain loss of subjects versus the initially estimated sample size. The main causes underlying such losses are death and increased physical or mental impairment of the participants.

## Discussion

There are studies in which a link has been established between cardiovascular risk factors and the development of dementia. However, few studies have related mild cognitive impairment to cardiovascular risk. We hope that this study will afford objective information on the representative prevalence of mild cognitive impairment in the population over 65 years of age in Salamanca.

We will conduct an analysis of the cardiovascular risk factors in the selected sample, allowing us to establish the possible correlation between certain cardiovascular risk markers and mild cognitive impairment. If found, such a correlation would be very useful for future studies designed to allow a certain prevention of such impairment, based on control of the cardiovascular risk factors.

The results obtained moreover will allow us to assess the predictive capacity in detecting cognitive impairment from the applied screening tests (MMSE and 7 Minute Screen), since their results will be contrasted with those of the detailed neuropsychological explorations. The importance of the early detection of cognitive impairment should be stressed, since it will allow the start of adequate treatment and thus can contribute to improve patient quality of life.

Lastly, we wish to point out that since a randomized population-based sample from the city of Salamanca is involved, the information obtained may be of increased representativeness - particularly considering that many studies suffer bias due to the lack of purely randomized criteria in selecting the participants.

## Competing interests

The authors declare that they have no competing interests.

## Authors' contributions

Conception of the idea for the study: MVP, RGG, ERS and VLF. Development of the protocol, organization and funding: MVP, ERS, RGG, VLF, SMS, CPA, TJA, CAC, MPM. Writing of the manuscript: MVP, ERS, RGG, VLF, and SMS. All the authors have read the draft critically, to make contributions, and have approved the final text.

## Pre-publication history

The pre-publication history for this paper can be accessed here:

http://www.biomedcentral.com/1471-2458/11/504/prepub

## References

[B1] Aguado-Ortego R Gómez-Carracedo A López-Arrieta J, García FG Trastornos psicopatológicos y conductuales en la demencia El anciano con demencia 2007 Madrid: Sociedad Española de Medicina Geriátrica 157 196

[B2] Ferri CP Prince M Brayne C Brodaty H Fratiglioni L Ganguli M Hall K Hasegawa K Hendrie H Huang Y Jorm A Mathers C Menezes PR Rimmer E Scazufca M Global prevalence of dementia: a Delphi consensus study Lancet 2005 366 9503 2112 2117 16360788 10.1016/S0140-6736(05)67889-0 PMC2850264

[B3] Impacto socio sanitario de las enfermedades neurológicas en España http://www.feeneurologia.com/

[B4] Antipsychotics for people with dementia BMJ 2008 337 a602 10.1136/bmj.a602 18614517 PMC2453249

[B5] Salzman C Jeste DV Meyer RE Cohen-Mansfield J Cummings J Grossberg GT Jarvik L Kraemer HC Lebowitz BD Maslow K Pollock BG Raskind M Schultz SK Wang P Zito JM Zubenko GS Elderly patients with dementia-related symptoms of severe agitation and aggression: consensus statement on treatment options, clinical trials methodology, and policy J Clin Psychiatry 2008 69 6 889 898 10.4088/JCP.v69n0602 18494535 PMC2674239

[B6] Utilización de antipsicóticos en pacientes ancianos con demencia Boletín Terapéutico Andaluz. Volume 25 200921216839

[B7] Schmidt R Schmidt H Fazekas F Vascular risk factors in dementia J Neurol 2000 247 2 81 87 10.1007/s004150050021 10751108

[B8] Lobo A Launer LJ Fratiglioni L Andersen K Di Carlo A Breteler MM Copeland JR Dartigues JF Jagger C Martinez-Lage J Soininen H Hofman A Prevalence of dementia and major subtypes in Europe: A collaborative study of population-based cohorts. Neurologic Diseases in the Elderly Research Group Neurology 2000 54 11 Suppl 5 S4 9 10854354

[B9] Staekenborg SS Su T van Straaten EC Lane R Scheltens P Barkhof F van der Flier WM Behavioural and psychological symptoms in vascular dementia; differences between small- and large-vessel disease J Neurol Neurosurg Psychiatry 2010 81 5 547 551 10.1136/jnnp.2009.187500 19965852

[B10] Thompson C Brodaty H Trollor J Sachdev P Behavioral and psychological symptoms associated with dementia subtype and severity Int Psychogeriatr 2010 22 2 300 305 10.1017/S1041610209991220 19906327

[B11] Luque-Santiago A de-Hoyos-Alonso MC Gorroñogoitia-Iturbe A Martín-Lesende I López-Torres-Hidalgo JD Baena-Díez JM Actividades preventivas en los mayores PAPPS Actualización 2009 Programa de actividades preventivas y de promoción de la salud 2009 Barcelona: semFYC 25 3821315641

[B12] Losada-Baltar A Izal-Fernández de Trocóniz M Montorio-Cerrato I Márquez-González M Pérez-Rojo G Eficacia diferencial de dos intervenciones psicoeducativas para cuidadores de familiares con demencia Revista de Neurología 2004 38 8 701 708 15122537

[B13] Livingston G Johnston K Katona C Paton J Lyketsos CG Systematic review of psychological approaches to the management of neuropsychiatric symptoms of dementia Am J Psychiatry 2005 162 11 1996 2021 10.1176/appi.ajp.162.11.1996 16263837

[B14] semFYC GdTdAFySdl Prescripción de ejercicio en el tratamiento de enfermedades crónicas 2006 Barcelona: semFYC

[B15] Launer LJ Andersen K Dewey ME Letenneur L Ott A Amaducci LA Brayne C Copeland JR Dartigues JF Kragh-Sorensen P Lobo A Martinez-Lage JM Stijnen T Hofman A Rates and risk factors for dementia and Alzheimer's disease: results from EURODEM pooled analyses. EURODEM Incidence Research Group and Work Groups. European Studies of Dementia Neurology 1999 52 1 78 84 9921852 10.1212/wnl.52.1.78

[B16] Berr C Wancata J Ritchie K Prevalence of dementia in the elderly in Europe Eur Neuropsychopharmacol 2005 15 4 463 471 10.1016/j.euroneuro.2005.04.003 15955676

[B17] Qiu C Winblad B Fratiglioni L The age-dependent relation of blood pressure to cognitive function and dementia Lancet Neurol 2005 4 8 487 499 10.1016/S1474-4422(05)70141-1 16033691

[B18] Duron E Hanon O Hypertension, cognitive decline and dementia Arch Cardiovasc Dis 2008 101 3 181 189 10.1016/S1875-2136(08)71801-1 18477946

[B19] Seux ML Thijs L Forette F Staessen JA Birkenhager WH Bulpitt CJ Girerd X Jaaskivi M Vanhanen H Kivinen P Yodfat Y Vanska O Antikainen R Laks T Webster JR Hakamaki T Lehtomaki E Lilov E Grigorov M Janculova K Halonen K Kohonen-Jalonen P Kermowa R Nachev C Tuomilehto J Correlates of cognitive status of old patients with isolated systolic hypertension: the Syst-Eur Vascular Dementia Project J Hypertens 1998 16 7 963 969 10.1097/00004872-199816070-00009 9794736

[B20] Starr JM Whalley LJ Inch S Shering PA Blood pressure and cognitive function in healthy old people J Am Geriatr Soc 1993 41 7 753 756 8315187 10.1111/j.1532-5415.1993.tb07466.x

[B21] Scherr PA Hebert LE Smith LA Evans DA Relation of blood pressure to cognitive function in the elderly Am J Epidemiol 1991 134 11 1303 1315 1755444 10.1093/oxfordjournals.aje.a116033

[B22] Farmer ME White LR Abbott RD Kittner SJ Kaplan E Wolz MM Brody JA Wolf PA Blood pressure and cognitive performance. The Framingham Study Am J Epidemiol 1987 126 6 1103 1114 3687920 10.1093/oxfordjournals.aje.a114749

[B23] Swan GE Carmelli D Larue A Systolic blood pressure tracking over 25 to 30 years and cognitive performance in older adults Stroke 1998 29 11 2334 2340 10.1161/01.STR.29.11.2334 9804644

[B24] Kilander L Nyman H Boberg M Lithell H The association between low diastolic blood pressure in middle age and cognitive function in old age. A population-based study Age Ageing 2000 29 3 243 248 10.1093/ageing/29.3.243 10855907

[B25] Whitmer RA Sidney S Selby J Johnston SC Yaffe K Midlife cardiovascular risk factors and risk of dementia in late life Neurology 2005 64 2 277 281 10.1212/01.WNL.0000149519.47454.F2 15668425

[B26] Kuller LH Lopez OL Newman A Beauchamp NJ Burke G Dulberg C Fitzpatrick A Fried L Haan MN Risk factors for dementia in the cardiovascular health cognition study Neuroepidemiology 2003 22 1 13 22 10.1159/000067109 12566949

[B27] Qiu C von Strauss E Fastbom J Winblad B Fratiglioni L Low blood pressure and risk of dementia in the Kungsholmen project: a 6-year follow-up study Arch Neurol 2003 60 2 223 228 10.1001/archneur.60.2.223 12580707

[B28] Skoog I Lernfelt B Landahl S Palmertz B Andreasson LA Nilsson L Persson G Oden A Svanborg A 15-year longitudinal study of blood pressure and dementia Lancet 1996 347 9009 1141 1145 10.1016/S0140-6736(96)90608-X 8609748

[B29] Hanon O Haulon S Lenoir H Seux ML Rigaud AS Safar M Girerd X Forette F Relationship between arterial stiffness and cognitive function in elderly subjects with complaints of memory loss Stroke 2005 36 10 2193 2197 10.1161/01.STR.0000181771.82518.1c 16151027

[B30] Verghese J Lipton RB Hall CB Kuslansky G Katz MJ Low blood pressure and the risk of dementia in very old individuals Neurology 2003 61 12 1667 1672 14694027 10.1212/01.wnl.0000098934.18300.be

[B31] Blood pressure lowering in patients without prior cerebrovascular disease for prevention of cognitive impairment and dementia Cochrane Database Syst Rev 2009 4 CD004034 10.1002/14651858.CD004034.pub3 PMC7163274 19821318

[B32] Kivipelto M Helkala EL Laakso MP Hanninen T Hallikainen M Alhainen K Soininen H Tuomilehto J Nissinen A Midlife vascular risk factors and Alzheimer's disease in later life: longitudinal, population based study BMJ 2001 322 7300 1447 1451 10.1136/bmj.322.7300.1447 11408299 PMC32306

[B33] Romas SN Tang MX Berglund L Mayeux R APOE genotype, plasma lipids, lipoproteins, and AD in community elderly Neurology 1999 53 3 517 521 10449113 10.1212/wnl.53.3.517

[B34] Kuusisto J Koivisto K Mykkanen L Helkala EL Vanhanen M Hanninen T Kervinen K Kesaniemi YA Riekkinen PJ Laakso M Association between features of the insulin resistance syndrome and Alzheimer's disease independently of apolipoprotein E4 phenotype: cross sectional population based study BMJ 1997 315 7115 1045 1049 9366728 10.1136/bmj.315.7115.1045 PMC2127678

[B35] Duron E Hanon O Vascular risk factors, cognitive decline, and dementia Vasc Health Risk Manag 2008 4 2 363 381 18561512 10.2147/vhrm.s1839 PMC2496986

[B36] Biessels GJ Staekenborg S Brunner E Brayne C Scheltens P Risk of dementia in diabetes mellitus: a systematic review Lancet Neurol 2006 5 1 64 74 10.1016/S1474-4422(05)70284-2 16361024

[B37] Xu W Qiu C Winblad B Fratiglioni L The effect of borderline diabetes on the risk of dementia and Alzheimer's disease Diabetes 2007 56 1 211 216 10.2337/db06-0879 17192484

[B38] Yaffe K Metabolic syndrome and cognitive disorders: is the sum greater than its parts? Alzheimer Dis Assoc Disord 2007 21 2 167 171 10.1097/WAD.0b013e318065bfd6 17545744

[B39] Vanhanen M Koivisto K Moilanen L Helkala EL Hanninen T Soininen H Kervinen K Kesaniemi YA Laakso M Kuusisto J Association of metabolic syndrome with Alzheimer disease: a population-based study Neurology 2006 67 5 843 847 10.1212/01.wnl.0000234037.91185.99 16966548

[B40] Dik MG Jonker C Comijs HC Deeg DJ Kok A Yaffe K Penninx BW Contribution of metabolic syndrome components to cognition in older individuals Diabetes Care 2007 30 10 2655 2660 10.2337/dc06-1190 17563341

[B41] Charlson ME Pompei P Ales KL MacKenzie CR A new method of classifying prognostic comorbidity in longitudinal studies: development and validation J Chronic Dis 1987 40 5 373 383 10.1016/0021-9681(87)90171-8 3558716

[B42] Charlson M Szatrowski TP Peterson J Gold J Validation of a combined comorbidity index J Clin Epidemiol 1994 47 11 1245 1251 10.1016/0895-4356(94)90129-5 7722560

[B43] Solomon PR Murphy CA Should we screen for Alzheimer's disease? A review of the evidence for and against screening Alzheimer's disease in primary care practice Geriatrics 2005 60 11 26 31 16287338

[B44] Sallis JF Haskell WL Wood PD Fortmann SP Rogers T Blair SN Paffenbarger RS Jr Physical activity assessment methodology in the Five-City Project Am J Epidemiol 1985 121 1 91 106 3964995 10.1093/oxfordjournals.aje.a113987

[B45] Mahoney FI Barthel DW Functional Evaluation: The Barthel Index Md State Med J 1965 14 61 65 14258950

[B46] Lawton MP Brody EM Assessment of older people: self-maintaining and instrumental activities of daily living Gerontologist 1969 9 3 179 186 10.1093/geront/9.3_Part_1.179 5349366

[B47] Jorm AF A short form of the Informant Questionnaire on Cognitive Decline in the Elderly (IQCODE): development and cross-validation Psychol Med 1994 24 1 145 153 10.1017/S003329170002691X 8208879

[B48] Jorm AF The Informant Questionnaire on cognitive decline in the elderly (IQCODE): a review Int Psychogeriatr 2004 16 3 275 293 10.1017/S1041610204000390 15559753

[B49] Folstein MF Folstein SE McHugh PR "Mini-mental state". A practical method for grading the cognitive state of patients for the clinician J Psychiatr Res 1975 12 3 189 198 10.1016/0022-3956(75)90026-6 1202204

[B50] Solomon PR Hirschoff A Kelly B Relin M Brush M DeVeaux RD Pendlebury WW A 7 minute neurocognitive screening battery highly sensitive to Alzheimer's disease Arch Neurol 1998 55 3 349 355 10.1001/archneur.55.3.349 9520009

[B51] del Ser Quijano T Sanchez Sanchez F Garcia de Yebenes MJ Otero Puime A Zunzunegui MV Munoz DG [Spanish version of the 7 Minute screening neurocognitive battery. Normative data of an elderly population sample over 70] Neurologia 2004 19 7 344 358 15273881

[B52] González-Montalvo JI Creación y validación de un test de lectura para el diagnóstico del deterioro mental en el anciano 1991 Universidad Complutense de Madrid

[B53] Wechsler D Wechsler Memory Scale - Revised manual 1987 San Antonio, TX: Psychological Corporation

[B54] Kaplan E Goodglass H Weintraub S Goodglass H, Kaplan E Test de vocabulario de Boston Evaluación de la afasia y trastornos relacionados 1986 Second Madrid: Panamericana

[B55] Goodglass H Kaplan E Evaluación de la afasia y de trastornos relacionados 1986 Madrid: Panamericana

[B56] Babcock H An experiment in the measurement of mental deterioration Archieves of Psychology 1930 117 105

[B57] Rey A Test de copia de la figura compleja 1987 Madrid: TEA

[B58] Benton AL TRVB. Test de Retención Visual de Benton 1981 Madrid: TEA

[B59] Dubois B Slachevsky A Litvan I Pillon B The FAB: a Frontal Assessment Battery at bedside Neurology 2000 55 11 1621 1626 11113214 10.1212/wnl.55.11.1621

[B60] Piatt AL Fields JA Paolo AM Troster AI Action (verb naming) fluency as an executive function measure: convergent and divergent evidence of validity Neuropsychologia 1999 37 13 1499 1503 10.1016/S0028-3932(99)00066-4 10617270

[B61] Reisberg B Ferris SH de Leon MJ Crook T The Global Deterioration Scale for assessment of primary degenerative dementia Am J Psychiatry 1982 139 9 1136 1139 7114305 10.1176/ajp.139.9.1136

